# Supplementary material for: Two-dimensional vacancy platelets as precursors for basal dislocation loops in hexagonal zirconium
Source: Nat Commun. 2020 Nov 13;11:5766. doi: 10.1038/s41467-020-19629-5 (PMC7666165; doi:10.1038/s41467-020-19629-5)
Supplement: Supplementary file 1 — Supplementary Information [file 41467_2020_19629_MOESM1_ESM.pdf]

Supplementary materials for

Two-dimensional vacancy platelets as precursors for basal dislocation loops in hexagonal zirconium

Si-Mian Liu[1], Irene J. Beyerlein[2], and Wei-Zhong Han[1,*]

[1]Center for Advancing Materials Performance from the Nanoscale, State Key Laboratory for Mechanical Behavior of Materials, Xi'an Jiaotong University, Xi'an 710049, China

[2] Mechanical Engineering Department, Materials Department, University of California, Santa Barbara, California 93106-5070, USA

The file includes:
Calculation of the energy of TVPs
Table S1
Table S2
Figs. S1-S5

Calculation of the energy of TVPs, one to six atomic layers thick

Plate-like voids along the basal plane are observed in Mg and Zr with the electron beam directed along the $[2\bar{1}\bar{1}0]$ direction [1-3]. High-resolution TEM images have provided evidence that a growing void has a thickness varying from 3 to 6 atomic layers [3]. Since the experimentally observed platelet vacancy cluster, or triangle vacancy plate (TVP), is found stable with only a few atomic layers, we calculated the energy of a TVP, considering thicknesses varying from 1 to 6 atomic layers. It is conjectured that the collapse of the TVP can produce a <c> dislocation loop of radius *R* once it attains a critical size, above which the energy of the loop becomes lower and is favored. The energy of a TVP as a function of l, the number of atomic layers, can be expressed as:

$$E_{TVP} = 2A_b\gamma_b + 3\,lA_l\gamma_l \tag{1}$$

where A_b is the area of basal plane, A_l is the lateral area per atomic layer of a thin vacancy TVP. The energies γ_b and γ_l are the surface energy of basal and lateral planes. For γ_b = γ_l, Eqn. (1) becomes

$$E_{TVP}(L) = \gamma_b(2\frac{\sqrt{3}}{4}L^2 + 3l\frac{Lc}{2}) \tag{2}$$

Considering the effect of hydrogen on the surface energy of basal plane [4,5], we adopt γ_s = $\gamma_l \approx$ 0.80 J/m^2 to calculate the formation energy of TVP. The relationship between *L* and *R* is $l\frac{\sqrt{3}}{4}L^2 = \pi R^2$, or $L = \sqrt{4\pi R^2/l\sqrt{3}}$. The volume of each vacancy in Zr is $\Omega = \frac{\sqrt{3}}{4}a^2c$ = 0.023 nm^3, where for Zr, *a* = 0.323 nm, *c* = 0.517 nm. The radius of a vacancy in Zr is $r_v = \sqrt[3]{\frac{3\Omega}{4\pi}} = 0.177\,nm$. The number of vacancies *n* in a <c> loop is $\frac{\pi R^2 c/2}{\Omega} \approx 35.3\ R^2$, thus $R = \sqrt{n/35.3}$. The comparison of the energy of <c> loop and TVP with 1 to 6 atomic layers is plotted in Fig. 4(c). We find that the energy of a three layered TVP crosses with the energy curves of <c> loop at 2$R_c \approx$ 13 nm and TVP at $L \approx$ 10 nm. This estimate for the critical size for the transformation of TVP into <c> loops is consistent with experimental observation.

There are several reports that indicate that the trace elements or alloying elements play a critical role in the nucleation of irradiation defects. In general, voids are more easily formed in alloys than in pure FCC metals [6]. For HCP metals, like Zr, less is known, but from the relatively few studies, the void formation under neutrons irradiation seems difficult without the assistance of

impurity gases or other alloy elements [7,8]. Several studies have attributed the formation of platelet or faceted voids in Zr after electron irradiation, to trace amounts of Fe [1,2]. Vacancy <c> loops are also mostly found in samples with impurity contents [8-10], another indication of their significant effect on vacancy effects. Atomistic calculations have shown that even 1ppm of solute Fe can decrease the formation energy of vacancy in Zr [10,11]. Hydrogen can also reduce the surface energy of basal plane in Zr and promote the aggregation of vacancies as well [4,5]. Other alloying elements, such as Sn, have been found to reduce the stacking fault energy for about 50% [12]. Impurities like oxygen also decrease the surface energy of voids in Ni and but cannot stabilize vacancy on basal plane in Zr [13,14]. The current energy-based estimate does not account for the kinetics and non-equilibrium conditions created by continuous ion bombardment. The formation of one to three layered TVPs is likely even in an ideally pure metal without the assistance of trace elements.

Table S1.

The impurities of Zr sample (wt.%).

Hf	Fe	Si	Sn	Ni	Cr	O	C
⩽0.001	⩽0.002	⩽0.001	⩽0.0005	⩽0.0007	⩽0.0008	⩽0.014	⩽0.001

Table S2.

The parameters associated with the $\vec{g}\cdot\vec{b}$ analysis used to determine the Burgers Vector. The vector $\overrightarrow{g}$= 0002 and $\vec{g}$ = $[1\bar{2}10]$ or $\vec{g} = [01\bar{1}0]$ was chosen for bright-field imaging to determine the <c> component of the dislocation loop. The invisibility rules for characterizing dislocation loops in Fig. S1 are used and listed below.

Number in Fig. S1	B	$10\bar{1}0$			$10\bar{1}1$		$2\bar{1}\bar{1}0$	Burgers Vector
	$\vec{g}$	0002	$\bar{1}2\bar{1}0$	$1\bar{2}10$	$\bar{1}101$	$1\bar{1}0\bar{1}$	$01\bar{1}0$	
1,2,3	$\vec{g}\cdot\vec{b}$	×	O	I	O	I	×	$\frac{1}{3}[\bar{2}110]$
4,5	$\vec{g}\cdot\vec{b}$	×	I	O	I	O	√	$\frac{1}{3}[1\bar{2}10]$

×-out of contrast; √-in contrast; I-inside contrast; O-outside contrast;

Figure S1.

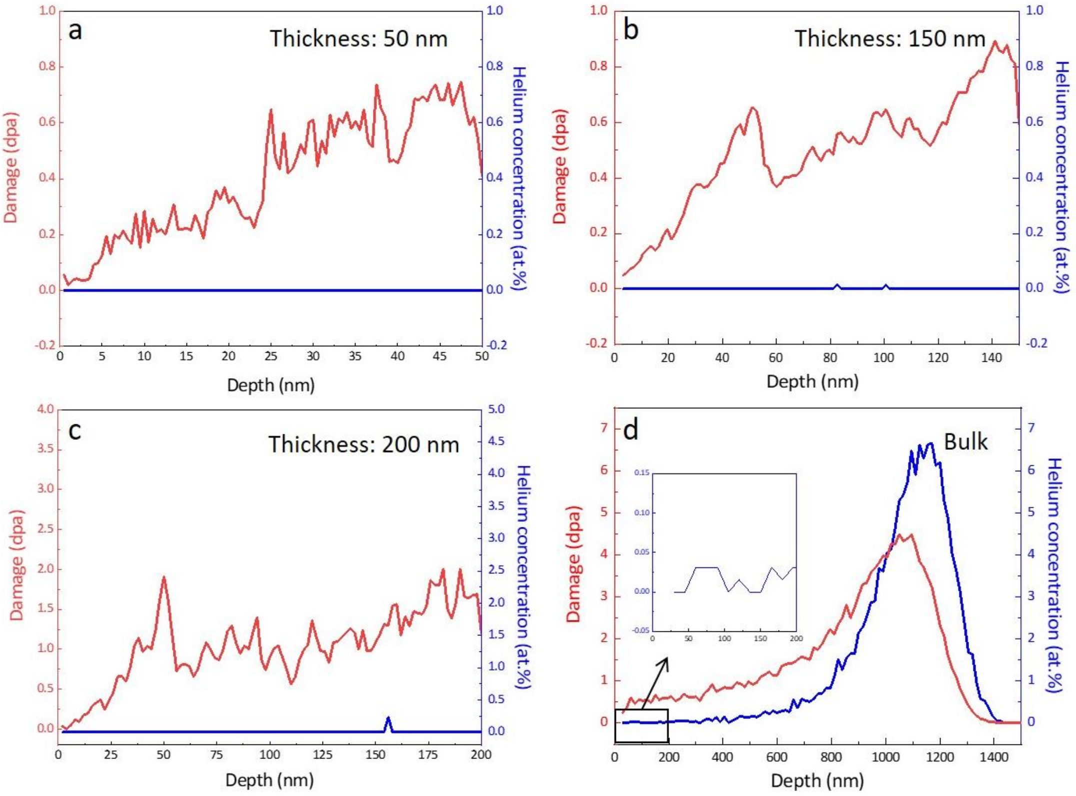

Fig. S1. The red and blue curves show the variation in radiation damage and helium concentration in the thin foil sample (a-c) with different thickness as well as in bulk (d). The SRIM calculation was conducted with a total number of 99999 helium ions. Except for the foil with 50 nm thickness, the foils with thickness of 150 nm and 200 nm all show a small helium concentration peak, which means just one or none helium in those thin foils sample. The small peak is likely just a statistic result of SRIM calculation. However, in the bulk implantation, helium concentration is not zero in the 0-200 nm range (d).

Figure S2.

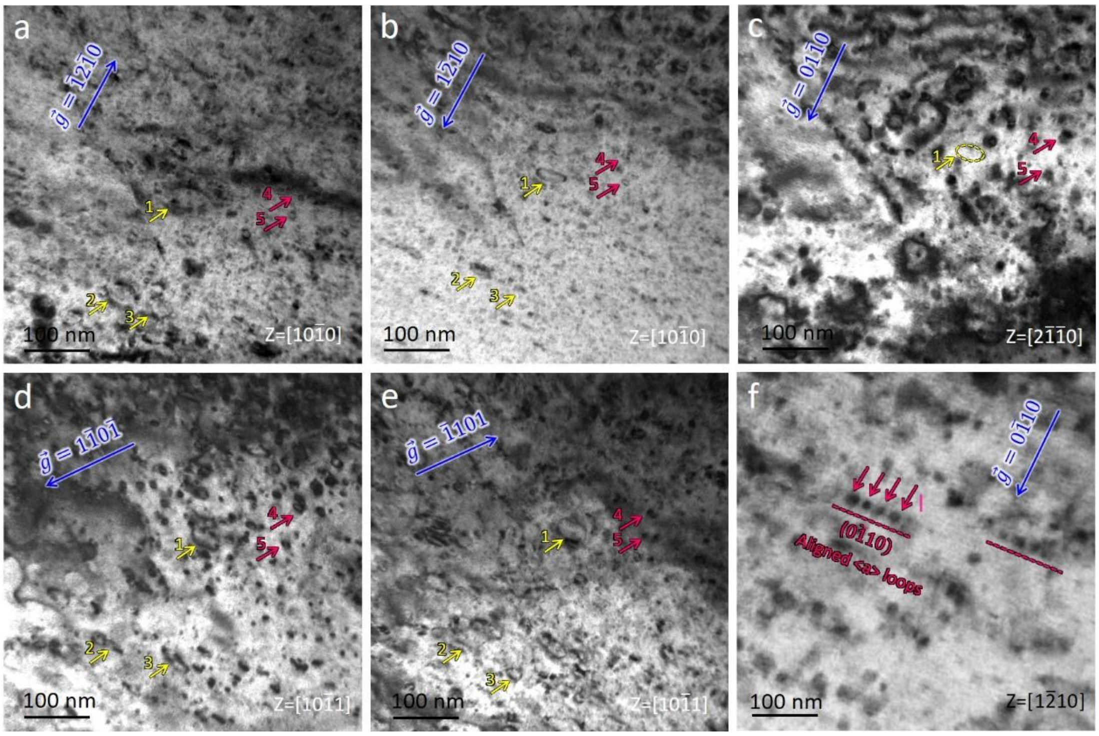

Fig. S2. (a-e) Determination of the properties of the dislocation loops in pure Zr irradiated by helium ions at 350C. (a) $\vec{g} = [1\bar{2}10], B = [\bar{1}010]$; (b) $\vec{g} = [\bar{1}2\bar{1}0], B = [\bar{1}010]$; (c) $\vec{g} = [01\bar{1}0], B = [\bar{2}110]$; (d) $\vec{g} = [1\bar{1}0\bar{1}], B = [\bar{1}01\bar{1}]$; (e) $\vec{g} = [\bar{1}101], B = [\bar{1}01\bar{1}]$; (f) Interstitial <a> dislocation loops form in a row parallel to the prismatic plane.

Figure S3.

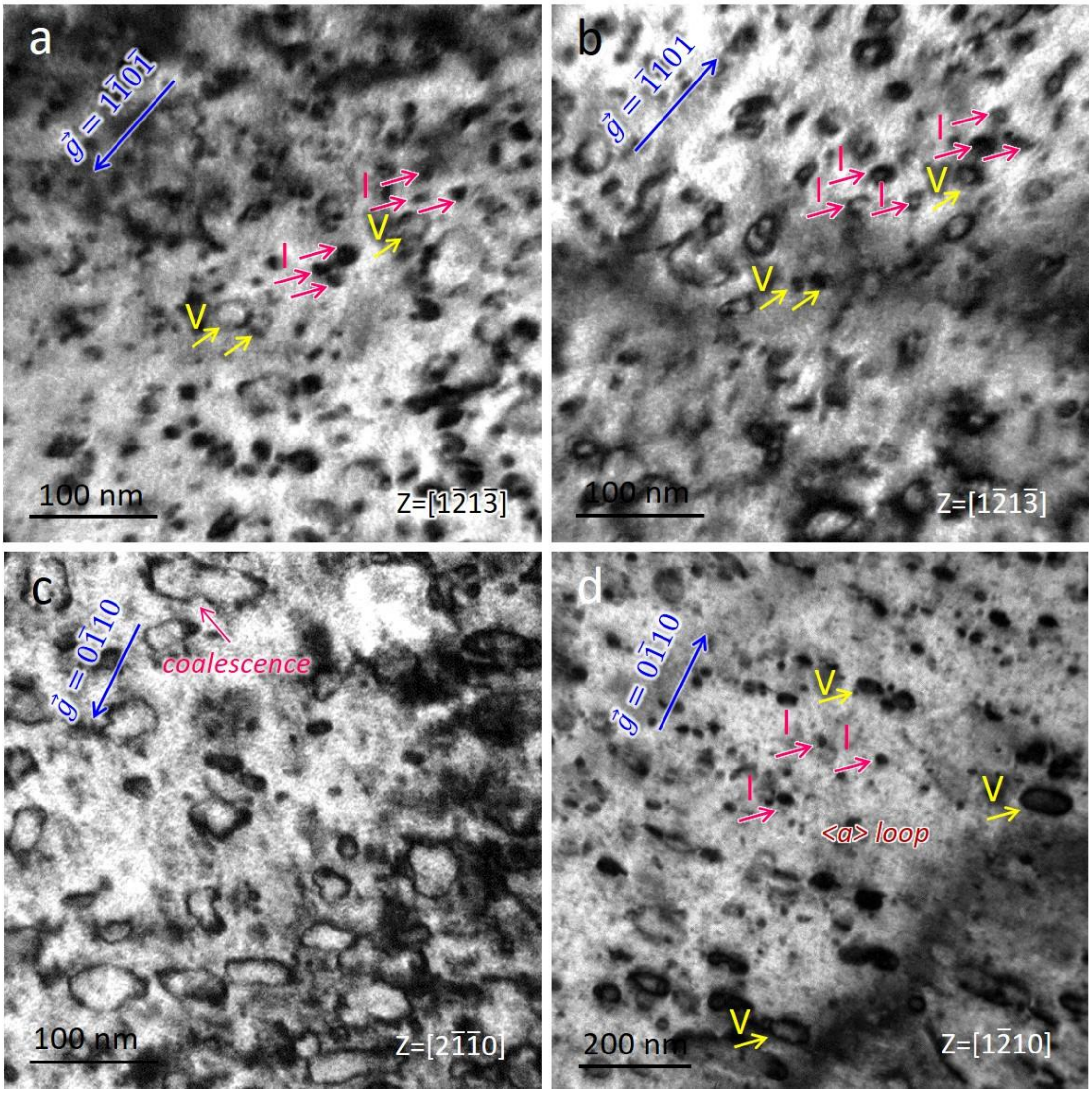

Fig. S3. (a) and (b) correspond respectively to the outside and inside contrast of the <a> dislocation loops under He^+ irradiation temperature at 400℃. (c) Coalescence of two <a> dislocation loops and formation of a large loop after He^+ implantation at 400℃. (d) The elliptical <a> dislocation loop has a long axis along the [0002] direction. The observation direction is along the $[\bar{2}110]$. The red and yellow arrows indicate interstitial and vacancy loops, respectively.

Figure S4

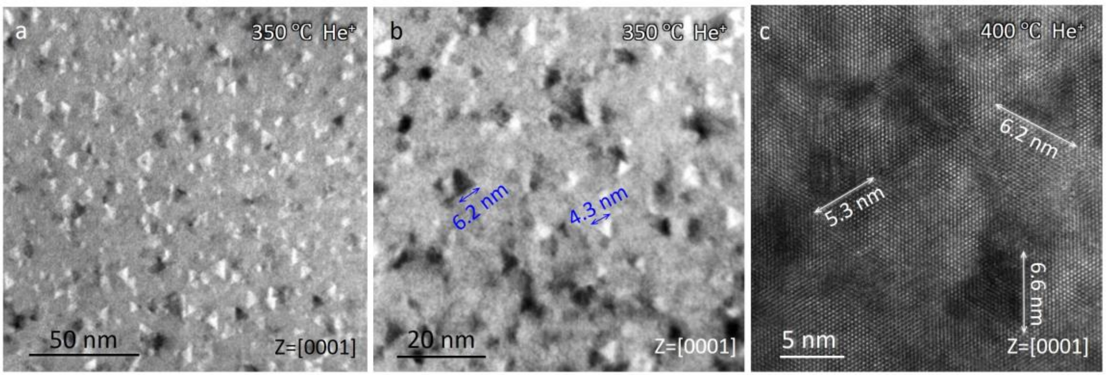

Fig. S4. Profuse formation of triangle-shaped defects on the basal plane in He^+ irradiated Zr (a) and (b). The images are taken with a defocus of -1000 nm. Most of the triangle-shaped defects have a white contrast, indicating most of them are vacancies clusters. (c) High-resolution TEM image of the triangle-shaped defects.

Figure S5

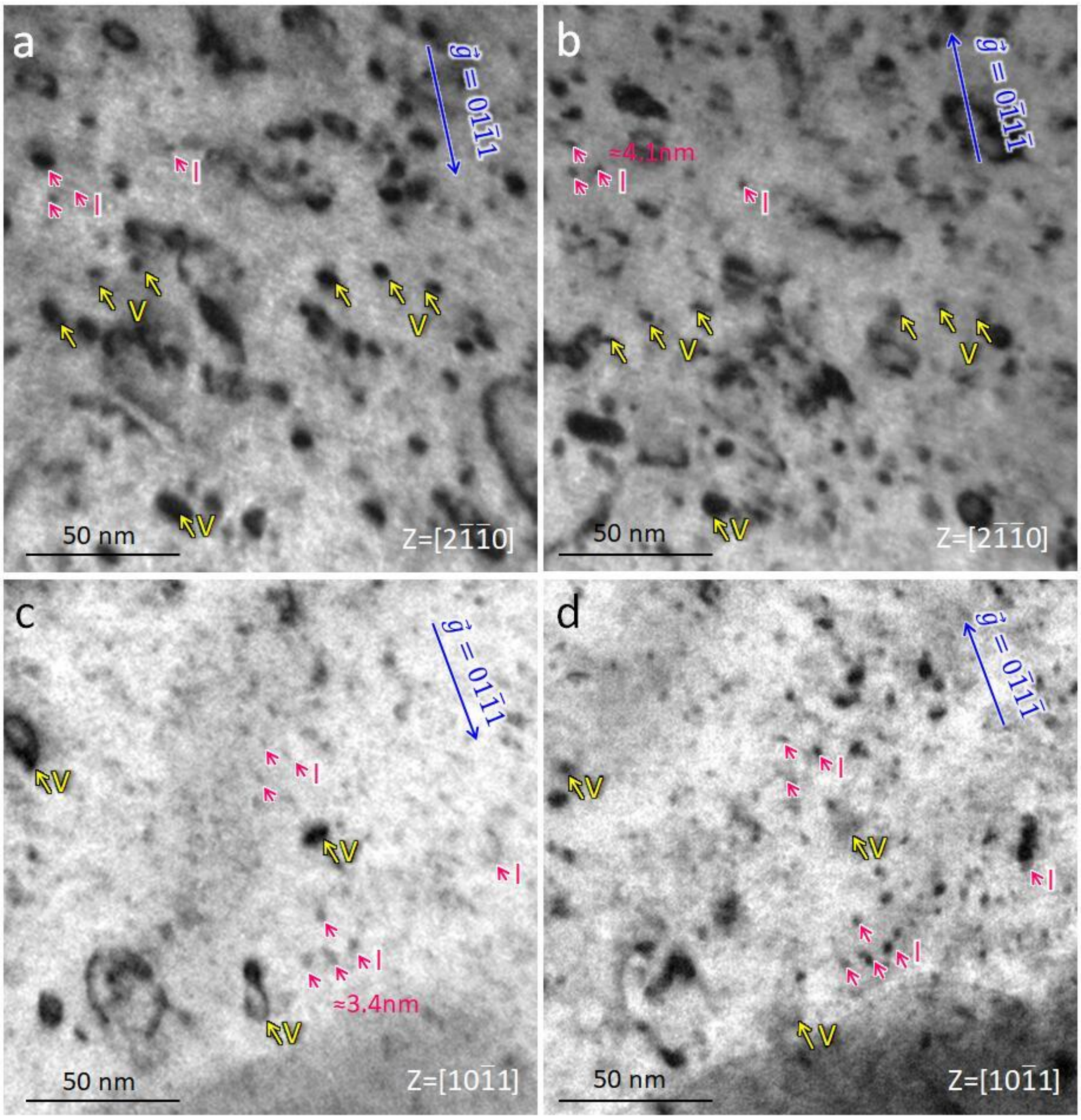

Fig. S5. The inside/outside contrast of tiny <a> dislocation loops with sizes smaller than 5 nm formed after He irradiation at 350℃. The red and yellow arrows mark the interstitial or vacancy loops, respectively.

References

[1] Griffiths, M. HVEM study of the effects of alloying elements and impurities on radiation damage in Zr-alloys. *J. Nucl. Mater.* **205**, 273-283 (1993).

[2] Griffiths, M., Styles, R.C., Woo, C.H., Phillipp, F. & Frank, W. Study of point defect mobilities in zirconium during electron irradiation in a high-voltage electron microscope. *J. Nucl. Mater.* **208**, 324-334 (1994).

[3] Xu, W. et al. In-situ atomic-scale observation of irradiation-induced void formation. *Nat. commun.* **4**, 2288 (2013).

[4] Maxwell, C.I., Torres, E. & Pencer, J. Molecular dynamics study of hydrogen-vacancy interactions in alpha-zirconium. *J. Nucl. Mater.* **511**, 341-352 (2018).

[5] Domain, C., Besson, R. & Legris, A. Atomic-scale ab initio study of the Zr-H system: II. Interaction of H with plane defects and mechanical properties. *Acta Mater.* **52**, 1495-1502 (2004).

[6] Ma, K., Décamps, B., Fraczkiewicz, A., Prima, F. & LoyerProst, M. Drastic influence of micro-alloying on Frank loop nature in Ni and Ni-based model alloys. *Mater. Res. Lett.* **8**, 201-207 (2020).

[7] Northwood, D.O., Gilbert, R.G., Neutron radiation damage in zirconium and its alloys. *Radiat. Eff.* **22**, 139-140 (1974).

[8] Griffiths, M. A review of microstructure evolution in zirconium alloys during irradiation. *J. Nucl. Mater.* **159**, 190-218 (1988).

[9] Griffiths, M. Evolution of microstructure in hcp metals during irradiation. *J. Nucl. Mater.* **205**, 225-241 (1993).

[10] Dai, C.et al. Primary damage production in the presence of extended defects and growth of vacancy-type dislocation loops in hcp zirconium. *Phys. Rev. Mater.* **3**, 043602 (2019).

[11] Pasianot, R.C., Pérez, R.A., Ramunni, V.P. & Weissmann, M. Ab initio approach to the effect of Fe on the diffusion in hcp Zr II: The energy barriers. *J. Nucl. Mater.* **392** 100-104 (2009).

[12] Udagawa, Y. Yamaguchi, M. Tsuru, T., Abe, H. & Sekimura, N. Effect of Sn and Nb on generalized stacking fault energy surfaces in zirconium and gamma hydride habit planes. *Philos. Mag.* **91**, 1665-1678 (2011).

[13] Seitzman, L.E., Wang, L.M. & Kulcinski, G.L. The effect of oxygen on void stability in nickel and austenitic steel. *J. Nucl. Mater.* **141–143**, 738–742 (1986).

[14] Christensen, M. et al. Effect of alloying elements on the properties of Zr and the Zr-H system. *J. Nucl. Mater.* **445**, 241-250 (2014).
